# Unweaving tangled mortality and antibiotic consumption data to detect disease outbreaks – Peaks, growths, and foresight in swine production

**DOI:** 10.1371/journal.pone.0223250

**Published:** 2019-10-09

**Authors:** Ana Carolina Lopes Antunes, Vibeke Frøkjær Jensen, Dan Jensen

**Affiliations:** 1 Division for Diagnostics & Scientific Advice–Epidemiology, National Veterinary Institute/Centre for Diagnostics–Technical University of Denmark, Lyngby, Denmark; 2 Department of Veterinary and Animal Sciences, University of Copenhagen, Frederiksberg C, Denmark; Lancaster University, UNITED KINGDOM

## Abstract

As our capacity to collect and store health data is increasing, a new challenge of transforming data into meaningful information for disease monitoring and surveillance has arisen. The aim of this study was to explore the potential of using livestock mortality and antibiotic consumption data as a proxy for detecting disease outbreaks at herd level. Changes in the monthly records of mortality and antibiotic consumption were monitored in Danish swine herds that became positive for porcine reproductive and respiratory syndrome (PRRS) and porcine pleuropneumonia. Laboratory serological results were used to identify herds that changed from a negative to a positive status for the diseases. A dynamic linear model with a linear growth component was used to model the data. Alarms about state changes were raised based on forecast errors, changes in the growth component, and the values of the retrospectively smoothed values of the growth component. In all cases, the alarms were defined based on credible intervals and assessed prior and after herds got a positive disease status. The number of herds with alarms based on mortality increased by 3% in the 3 months prior to laboratory confirmation of PRRS-positive herds (Se = 0.47). A 22% rise in the number of weaner herds with alarms based on the consumption of antibiotics for respiratory diseases was found 1 month prior to these herds becoming PRRS-positive (Se = 0.22). For porcine pleuropneumonia-positive herds, a 10% increase in antibiotic consumption for respiratory diseases in sow herds was seen 1 month prior to a positive result (Se = 0.5). Monitoring changes in mortality data and antibiotic consumption showed changes at herd level prior to and in the same month as confirmation from diagnostic tests. These results also show a potential value for using these data streams as part of surveillance strategies.

## Introduction

Monitoring systems are essential for detecting changes in disease status in a timely and effective manner [1]. This is of paramount importance to reduce the impact of outbreaks and to avoid trade restrictions. The ability to detect changes in disease occurrence depends to a large extent upon the choice of data source [2].

The increasing availability of electronic records collected actively or passively enables the targeting of specific groups and the monitoring and prediction of specific events [3–5]. It is believed that using large volumes of data will help to improve the timeliness of epidemiological information, resulting in more accurate disease surveillance [6].

Data relating to the livestock population demography, productivity, and health are collected on a continuous basis worldwide [7]. As our capacity to collect and store these data rapidly increases, a new challenge of transforming the data into meaningful information about animal health for disease monitoring is arising [8–10]. In Denmark, the current governmental and industry-owned databases cover different aspects of animal health data, including changes in infectious disease status in terms of endemic disease in subpopulations, antibiotic usage, and mortality [11]. All farmers within the European Union are obliged to send animal carcasses to rendering plants for food safety and traceability purposes [12], which ensures a continuous data flow of mortality data.

There has been an increasing concern about the association between the use of antibiotics in livestock animals and the emergence of antibiotic resistance [13]. The VetStat database was implemented in Denmark to monitor the use of drugs in livestock and to ensure transparency and compliance with legislation [14]. It is mandatory to register all purchases of prescription-only drugs for production animals either passively (by pharmacies and feed mills at the point of sale) or actively (by veterinarians).

Further research into translating these types of data into meaningful information for disease monitoring is needed [15]. Previous studies have demonstrated the potential use of mortality data for this purpose [16, 17]. An association has also been shown between disease occurrence and an increase in antibiotic use in Danish swine herds [18–20]. However, the potential use of these data for outbreak detection and surveillance purposes at herd level remains unexplored. One possible approach is to train models on data from healthy herds, set up a monitoring system based on these model and, any deviation from the expected outcome sets a warning, as described in previous studies [21–23]. This is particularly useful for authorities to detect and monitor outbreaks and follow up changes in prevalence on the national scale. Although farmers might identify the presence of disease outbreaks based on clinical symptoms and laboratory diagnostic tests, the Danish Veterinarian authorities do not have access to these data, due to the legal restrictions regarding data protection in Europe and in Denmark.

The aim of this study was to explore the potential use of mortality data and antibiotic consumption data for detecting outbreaks, assuming that in cases of outbreaks, an increase in mortality and antibiotic consumption, and a change in disease health status will occur in quick succession.

Changes in mortality data and antibiotic consumption at herd level were monitored in Danish swine herds that became positive for Porcine Reproductive and Respiratory Syndrome (PRRS) and porcine pleuropneumonia (caused by *Actinobacillus pleuropneumoniae*).

## Materials and methods

In this study, we worked with three different age groups of pigs: weaners (up to 30 kg), finishers (>30 kg—slaughter), and sows (including gilts). The data for each age group were processed and modeled separately. Each age group within a farm is referred to as ‘a herd’ throughout the manuscript. Mortality data were analyzed by herd due to physiological differences: higher mortality can be expected in weaners compared to sows and finishers due to disease, thermal stress, and nutrition [24]. The amount of antibiotics prescribed in Danish swine farms also differs across the three age groups [25]. All analysis, modeling, and representations of the data were done using the R software (version 3.3.3) [26], a language and environment for statistical computing. The R codes used in this study can be found in S1 Appendix.

### Laboratory data

Laboratory submission data stored in information management systems in the National Veterinary Institute–Technical University of Denmark (DTU Vet) were used in this study to define the time at which infections were confirmed for a given herd. Individual herds were tracked using data from the Central Husbandry Register (CHR). All individual blood samples collected on the same day from different animals from a given farm were processed as a single laboratory submission. For this study, we only included submissions with at least ten individual blood samples serologically tested for surveillance and monitoring purposes. These serological tests were either Blocking Enzyme-Linked Immunosorbent Assays (ELISA) [27, 28] and/or Immunoperoxidase Monolayer Assays (IPMA) [29] for PRRS type 1 and type 2, or an in-house indirect ELISA and Complement Fixation Test [30–33] for porcine pleuropneumonia (caused by *Actinobacillus pleuropneumoniae*) serovars 1, 2, 3, 4, 5, 6, 7, 8, 9, 10, 11, and 12. No distinction was made between the different PRRS types and porcine pleuropneumonia serotypes, with each herd considered to be positive for a given disease if at least one of the types or serotypes tested positive. At least 2 positive blood samples per submission was needed to be considered a positive herd. The month in which a herd obtained a positive diagnostic test result, which was preceded by negative diagnostic test results for a minimum period of 2 months, was used as a reference time point for comparing temporal changes on mortality and antibiotic consumption records prior, during and after changes in herd disease status.

### Mortality data

Mortality data from December 2013 to September 2017 were provided by the Danish Veterinary and Food Administration, which is the entity responsible for estimating swine mortality on all Danish farms. The estimation is based on information from the CHR and the Swine Movement Database (SMD), both owned by the Veterinary and Food Administration. The CHR is the national database of all Danish farm demographics, where a farm is defined as a single location with its own CHR number. The SMD includes information on all movements of swine from different herds in Denmark. The date and number of dead finishers and/or sows transported from a farm to the rendering plant are registered in the SMD. Regarding weaners, the number of small and medium containers (with room for seven and nine weaners, respectively) is registered. Movements from farms to rendering plants registered in the SMD are used to estimate the number of dead animals for each farm.

The monthly mortality rate was calculated as a proportion: the number of dead animals in a given herd registered within a given month, divided by the number of animals within the same herd recorded in the CHR as being present on the farm. We have referred to this proportion as ‘mortality’ throughout the manuscript. In order to include only active farms in the study, sow and finisher herds with zero mortality in any 6 consecutive months were excluded from the analysis. Weaner herds with zero mortality in 2 consecutive months were also excluded from the analysis. The data were further cleaned by deleting entries from herds with any negative records of mortality (< 0.1% of the records).

### VetStat data

All purchases of antibiotics made between September 2013 and December 2017 for herds that shifted from a negative to positive status for PRRS and porcine pleuropneumonia were included in the analysis. For each purchase, the date, CHR number, target species, age group, and disease group at the time of prescription were recorded. Internal validation showed inconsistency between herds and species in less than 1% of records for the included farms. These errors were corrected when possible, based on a comparison of species registered on the farm and information on the prescription record (which included species, age group, and medicinal product). The data were further cleaned by deleting the negative records of antibiotic purchases along with their corresponding positive records, because a lag of weeks or even months occurs. Negative records occur when prescribed medicines are not collected at the pharmacy.

To make comparisons among the herds, we used Animal Daily Doses (ADD) as the unit of measurement for antibiotic use [34]; ADD_kg_ defines, for a given medicinal product, the dose of treatment for 1 kg animal bodyweight. In this study, the ADDs defined by the Veterinary and Food Administration were applied. The monthly amount of antibiotics consumed per animal within a herd (ADD_kg_/pig*days _herd, month_) was calculated for each herd as described by Vigre *et al*. [19].

In summary, the number of days between consecutive purchases was used to estimate the average daily use of antibiotics within the herd, assuming that all purchased antibiotics were used at a constant rate. When the time between two consecutive purchases was more than 90 days, the prescribed medicine was assumed to be used at a constant rate within 90 days of prescription; the daily amount was calculated by dividing the total amount purchased by 90 days. The data were then aggregated per month and the number of swine registered in the CHR was used to calculate the amount of antibiotic usage per pig-day for each herd. Unlike the approach used by Vigre *et al*. [19], the average ADD_kg_/(pig*days) _herd_ from previous purchases for each herd was used to calculate the length of time from the last recorded purchase. Additionally, the standard dose for each herd–ADD_kg_/(pig*days) _herd_ per month–was then divided by an assumed average live weight of 50 kg for finishers, 200 kg for sows, and 15 kg weaners [34], corresponding to a conversion of ADD_kg_ to standard dose ADD for animals in each herd. These values were calculated for the total amount of antibiotic used (i.e. including all disease groups), for antibiotics purchased for both the respiratory disease group and for the reproductive and urogenital group.

### Learning set and test set

Herds with at least two data streams (time-series of mortality and antibiotic consumption for any disease group) were considered to be active herds (i.e. herds with mortality records of > 0) and were included in the study. The herds were initially divided into a healthy set and a non-healthy set. For a herd to be considered healthy, it needed to match all of the following criteria: 1) it should be tested for PRRS and porcine pleuropneumonia with at least three laboratory submissions within the study period, 2) it should have a maximum of 12 months between consecutive submissions, and 3) it should have only negative diagnostic serology results for both of the diseases considered in this study.

To be included as a non-healthy herd, a herd should have been tested for PRRS and porcine pleuropneumonia, and further had to match the following criteria: 1) at least three submissions with a maximum of 12 months between each, 2) only one shift from negative to positive status for a given disease within the study period, and 3) 10 months of mortality and antibiotic consumption (total and for the different disease groups) data prior to becoming positive for a given disease. All herds which did not meet either set of criteria were excluded from the current study.

The healthy herds were randomly divided into a learning set and a healthy test set. The learning set included 10% of the healthy herds for each age group (i.e. weaners, sows and finishers). The remaining 90% of the herds of each age group were used as the healthy test set. The non-healthy herds were used directly as the non-healthy test set.

The learning set was used to define the initial prior distributions and the variance components of the models, as described below. This was done to ensure that the models would be optimized for forecasting the data streams under the assumption that the herd is healthy. Large deviations between forecasted and observed values could then be taken as an indication that the herd was deviating from the healthy state. This approach to defining the DLM has been described in previous studies ([23, 35, 36]).

The healthy test set was used to estimate the specificity of various applications of the model on the various age groups, while the non-healthy test set was used to estimate the block-sensitivity of the same applications of the model on the various age groups, as described in detail below.

### Modeling and parameterization

A univariate dynamic linear model (DLM) with a local linear trend component, as described in detail by West and Harrison [37], was used to model each data stream at herd level. The general aim of a DLM is to estimate the underlying true value of a given variable, which might be expected to change over time. This is achieved by sequentially applying a Bayesian framework in which the observed data (i.e. mortality or antibiotic consumption) are combined with available prior information, which is expressed as a prior distribution at each time step. A conditional distribution is estimated for each time step, which is used to express the estimated underlying true level of the modeled system. These models can be used to produce one-step forecasts of the variable of interest, which can then be compared with the observed data.

In general, the DLM is defined by a set of two equations, namely the observation equation (Eq 1) and the system equation (Eq 2):

$$Y_{t}=F^{\prime }\theta_{t}+v_{t},\;v_{t}∼N(0,V)$$ (1)

$$\theta_{t}=G\theta_{t-1}+w_{t},\;w_{t}∼N(0,W_{t})$$ (2),

where Eq 1 describes how the observed values depend on the underlying parameter vector (***θ***_*t*_) and Eq 2 describes the systematic evolution of the parameter vector from time *t*-1 to *t*. The variance components (*V* and ***W***_*t*_) are referred to as the observational variance and system variance, respectively.

Our model included a linear growth component consisting of a time-varying slope, i.e. a local linear trend. This local linear trend was incorporated into the model to allow the system to adapt to a possible positive or negative growth in mortality or antibiotic consumption. This inclusion was achieved by making the parameter vector a column vector with a length of 2:

$$\theta_{t}=\left[\begin{array}{c} m_{t}\\ T_{t} \end{array}\right]$$ (3),

where *m*_*t*_ is the filtered mean of the mortality or antibiotic consumption at time *t* and *T*_*t*_ is the local linear trend at time *t*. If *T*_*t*_ deviates from 0 then the *m*_*t*_ systematically increases or decreases over time, depending on whether *T*_*t*_ is positive or negative, respectively. The prior variance of the parameter vector at time *t*, ***C***_*t*_, is a 2x2 matrix with the following structure:

$$C_{t}=[\begin{array}{c} C_{t}[1,1]\;C_{t}[1,2]\\ C_{t}[2,1]\;C_{t}[2,2] \end{array}]$$ (4),

where *C*_*t*_[1,1] is the prior variance of *m*_*t*_, *C*_*t*_[2,2] is the prior variance of *T*_*t*_, and *C*_*t*_[1,2] = *C*_*t*_[2,1] is the prior co-variance between *m*_*t*_ and *T*_*t*_. The initial prior distribution *D*_0_~[*θ*_0_,*C*_0_] for the DLM was estimated for each data type, i.e. mortality and antibiotic consumption: *m*_0_ was estimated as the average value of all observations of the relevant data type in the learning set, while the initial growth component, *T*_0_ was set to 0. The prior variance of the mean, *C*_0_[1,1], was defined as 10% of *m*_0_, the prior variance of the local linear trend, *C*_0_[2,2], was defined as 1% of *m*_0_, and the prior co-variance, *C*_0_[1,2] = *C*_0_[2,1], was set to 0. The various elements of *m*_t_ and *C*_t_ underwent Bayesian updating at each time step via the Kalman filter, which has been described in detail by West & Harrison [37].

The transposed design matrix (***F***′) had the following structure:

$$F^{\prime }=[\begin{array}{cc} 1 & 0 \end{array}]$$ (5),

while the system matrix (***G***) was given the following structure:

$$G=\left[\begin{array}{cc} 1 & 1\\ 0 & 1 \end{array}\right]$$ (6)

This model is designed to account for a local linear trend and follows the examples of previous works, e.g. [38] and [36]. The values of the parameter vector were generally updated using the Bayesian framework of the Kalman filter, as previously described [37]. For all observations of the value 0, however, the parameter vector would be updated deterministically in accordance with Eq 2, omitting the Bayesian aspects of the update.

At each time step, the forecast error is calculated as the observed value minus the forecasted value. Furthermore, the forecast errors *e*_*t*_ were standardized with respect to the forecast variance, Q_t_, such that $u_{t}=e_{t}/\sqrt{Q_{t}}$.

In our study, the observational variance (*V*) was kept constant, while the system variance (***W***_*t*_) was estimated continuously. Both the observational variance and the system variance were optimized based on each training set. The system variance was approximated using a discount factor (δ), which can take a value between 0 and 1. The discount factor directly expresses the decay of information, in the sense that high values (close to 1) imply a small system variance with very slow adaptation to the observations. Low values (i.e. close to 0) on the other hand leads to a very adaptive model where the filtered mean fluctuates more with the data. Different combinations of values for *V* and δ were tested to optimize the models using the learning set. Specifically, values of *V* ranging from 0.0001 to 0.001 by increments of 0.0001 (mortality as proportion) were evaluated for the mortality data, and values of *V* ranging from 0.1 to 6 in increments of 0.1 (ADD_kg_/(pig*days) _herd_ per month) were evaluated for the antibiotic consumption (total and disease group) data. Similarly, values of δ ranging from 0.1 to 1 by steps of 0.01 were tested for both mortality and antibiotic consumption data. The combination of δ and *V* that minimized the sum of the squared forecast errors, and where the standardized forecast most closely followed a standard normal distribution, was chosen as the final variance parameters of the respective models.

The adaptive coefficient (*A*_t_, calculated as part of the Kalman filter) and the filtered local linear trend values obtained from the models for the different training sets were visually assessed to determine the burn-in period of the DLM.

The computational stability of the DLM, with respect to the initial prior distribution, was assessed by repeatedly (N = 20) initiating the DLM with a randomly chosen initial prior distribution. In each repetition, a random value for *m*_0_ was taken from a uniform distribution ranging from the lowest to the highest values observed for each data type in the learning set, and the initial prior distribution was defined from this value as described above. Subsequently, the mean absolute deviations (MAD) between the filtered means of the optimized models and the filtered means from the randomly initiated models, were calculated for each time step.

A smoothing filter for retrospective analysis of time series data has been described in detail by West & Harrison [37]. This smoothing filter combines the prospectively filtered values made using the DLM with the observed values, in order to further reduce noise in the time series data in a stepwise and retrospective manner. This smoothing algorithm is sometimes used as part of the expectation-maximization algorithm to estimate constant variance parameters for a DLM, as described by various authors [23, 36, 39]. In our study, however, retrospective filtering was applied to the different time series to perform a retrospective analysis of the data in order to detect shifts in the level. The smoothing filter with a linear growth component described by West & Harrison [37] was applied to the outputs from the DLM: the smoothened distribution at each time *t* is calculated as:

$$({ϴ}_{t-k}|D_{t})∼N[a_{t}(-k),R_{t}(-k)],\;k\geq 0$$ (7)

$$C({ϴ}_{t-k-j},{ϴ}_{t-k}|D_{t})=A_{t-k-j,t-k}R_{t}(-k),\;j\geq 0$$ (8)

where *a*_t_ represents the prior mean, *R*_t_ is the prior variance, and *A*_t_ is the adaptive coefficient.

### Specificity

The specificities of the monitoring methods (based on the DLM forecast errors, DLM growth component and the smoothed growth component) were estimated using the healthy test set. The specificity was defined as the percentage of months with no alarms out of the total number of observations for each healthy test set between January 2014 and September 2017 (total of 46 observation-months). This per-observation based definition of specificity is common practice within precision livestock farming when monitoring time series of data for detection of undesired events [40, 41].

### Block sensitivity

The sensitivities of the various monitoring methods were estimated using the non-healthy test set. For each herd, we considered a time window ranging from -4 months to +4 observations (inclusive) relative to the time where a given disease was confirmed from a positive serological test. If at least one alarm was raised by a given monitoring method during this time window the time block was considered to have a true positive alarm. The block sensitivity of a given monitoring method was thus calculated as the percentage of non-healthy herds, which had at least one alarm within ±4 months (i.e. ±4 observations) of a positive serological tests. The use of block sensitivity is common practice in the precision livestock science [40, 41].

### Youden’s J index

To assess and compare the performance of the monitoring methods, Youden’s J index [42] was calculated for each method and age group, according to Eq 9:

J=Sp+Se−1 (9),

where Sp and Se are the specificity and block sensitivity, respectively, as described above.

### Monitoring changes in data streams

Changes in the data were monitored using Shewhart control charts [43], based on 2σ and 3σ upper control limits, applied to DLM forecasting errors. This method has a baseline calculated based on the average of the all observations available before a given time period (*t*-1) and makes it a robust method to detect large shifts in the process mean or variance [43]. The forecast errors were monitored at herd level and for each data stream.

For both the DLM and the smoother, the growth was extracted from the θ vector for each time step *t*. The variance of the trend parameter was calculated from the variance-covariance matrix for the posterior distribution, as previously described [44]. This variance was used to calculate 95% and 99% credible intervals (CI). Alarms were generated if the trend was significantly above zero, given a 95% CI and a 99% CI.

The different test sets (i.e. data from non-healthy herds) were included in the analysis and assumed to be mutually independent. The data were analyzed for a given month, with the month in which herds had a change in disease status used as the reference month (month 0). The months before and after the reference month will be referred to as “prior” and “after” throughout the manuscript.

The percentage of herds with alarms for a given month *i* was calculated as:

$${\%of\;active\;herds\;with\;alarms}_{i}=\frac{\sum x_{i}}{{n.herd}_{i}}∙100\%$$ (10)

where *x*_*i*_ is the number of herds with alarms for a given month *i* and *n*.*herd*_*i*_ corresponds to the total number of active herds for month *i*, for a given data stream and monitoring method.

This percentage of herds with alarms for a given month and the block sensitivity described above, serve to complement each other, thus providing a more detailed understanding of the alarms in terms of both timeliness and reliability.

**Fig 1:**
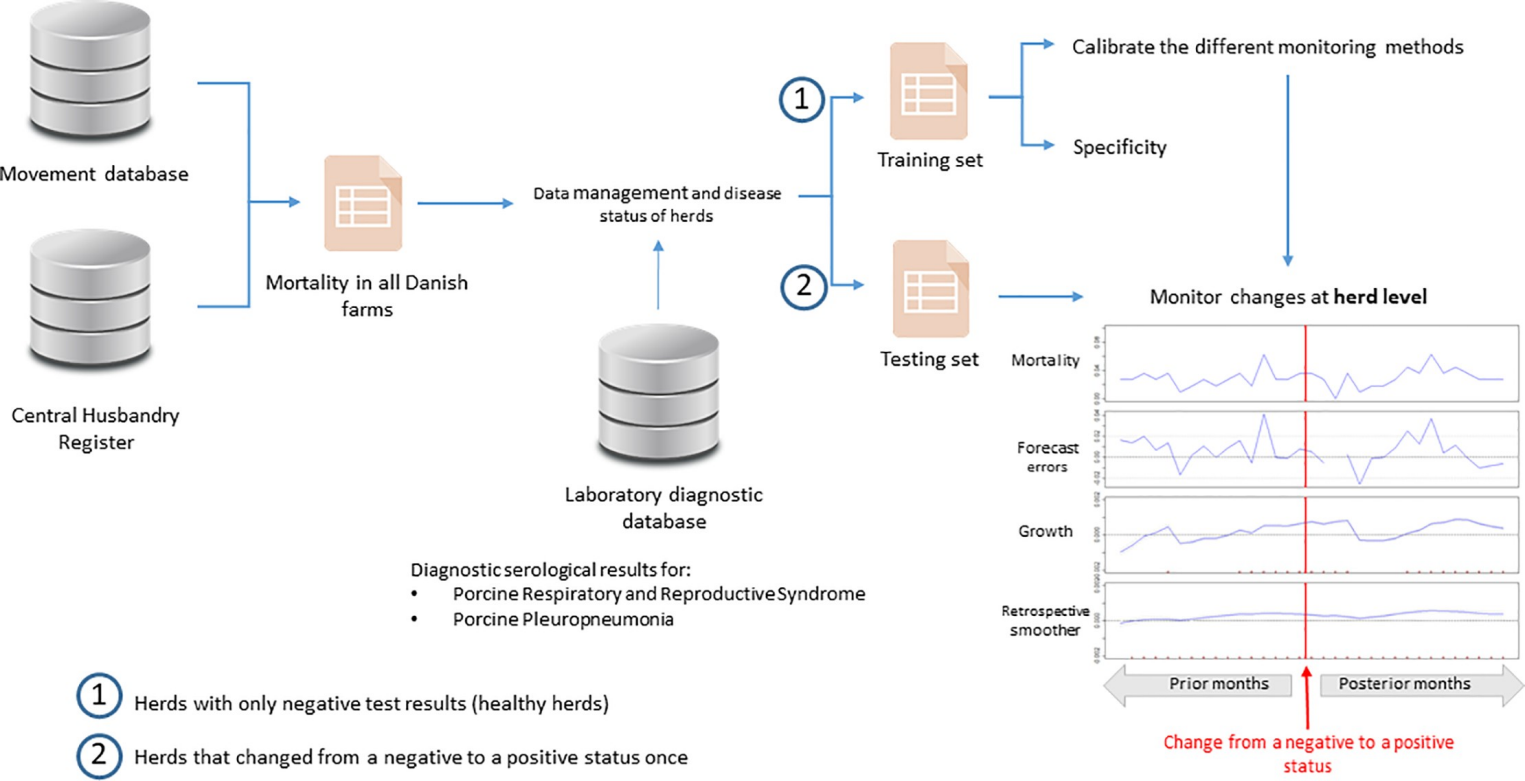
Schematic representation of the data sources, data management, and analysis for monitoring changes in mortality data.

All data processing, analysis and modelling is outlined schematically in Fig 1 using mortality data as an example.

## Results

### Descriptive statistics and model parameters

Table 1 shows the number of healthy and non-healthy herds included in the study as learning, training and test datasets. Table 2 has the initial prior distribution for each data stream and herd type.

**Table 1:** Number of herds included in the healthy and non-healthy sets.

	Herd	Mortality (no. of herds)	Ab total^1^ (no. of herds)	Ab resp^2^ (no. of herds)	Ab repro^3^ (no. of herds)
Healthy herds*	Weaner	110	34	7	-
	Sow	112	47	18	45
	Finisher	108	25	-	-
Test set PRRS	Weaner	35	22	9	-
	Sow	30	24	11	48
	Finisher	36	17	2	-
Test set Porcine pleuropneumonia	Weaner	21	19	4	-
	Sow	20	20	12	-
	Finisher	20	12	4	-

PRRS: Porcine Reproductive and Respiratory Syndrome

*Total number of herds included in the learning and training sets.

^1^Ab total: Total antibiotic consumption

^2^Ab resp: Antibiotic consumption for respiratory diseases

^3^Ab repro: Antibiotic consumption for reproductive and urogenital diseases

**Table 2:** The initial prior distributions for each herd and data stream, as estimated from the learning data.

Data	Herd	*θ*_0_	*C*_0_
Mortality	Weaner	$\left[\begin{array}{c} 0.0642\\ 0.0000 \end{array}\right]$	$\left[\begin{array}{cc} 0.0064] & 0.0000\\ 0.0000 & 0.0006 \end{array}\right]$
	Sow	$\left[\begin{array}{c} 0.0092\\ 0.0000 \end{array}\right]$	$\left[\begin{array}{cc} 0.00092 & 0.00000\\ 0.00000 & 0.00009 \end{array}\right]$
	Finisher	$\left[\begin{array}{c} 0.0212\\ 0.0000 \end{array}\right]$	$\left[\begin{array}{cc} 0.0021 & 0.0000\\ 0.0000 & 0.0002 \end{array}\right]$
Total antibiotic consumption	Weaner	$\left[\begin{array}{c} 3.387\\ 0.0000 \end{array}\right]$	$\left[\begin{array}{cc} 0.3387 & 0.0000\\ 0.0000 & 0.0339 \end{array}\right]$
	Sow	$\left[\begin{array}{c} 0.4170\\ 0.0000 \end{array}\right]$	$\left[\begin{array}{cc} 0.0417 & 0.0000\\ 0.0000 & 0.0042 \end{array}\right]$
	Finisher	$\left[\begin{array}{c} 0.5029\\ 0.0000 \end{array}\right]$	$\left[\begin{array}{cc} 0.0503 & 0.0000\\ 0.0000 & 0.0050 \end{array}\right]$
Antibiotic consumption for respiratory diseases	Weaner	$\left[\begin{array}{c} 0.4318\\ 0.0000 \end{array}\right]$	$\left[\begin{array}{cc} 0.0432 & 0.0000]\\ 0.0000 & 0.0043 \end{array}\right]$
	Sow	$\left[\begin{array}{c} 0.2805\\ 0.0000 \end{array}\right]$	$\left[\begin{array}{cc} 0.0281 & 0.0000\\ 0.0000 & 0.0028 \end{array}\right]$
Antibiotic consumption for reproductive and urogenital diseases	Sow	$\left[\begin{array}{c} 3.2211\\ 0.0000 \end{array}\right]$	$\left[\begin{array}{cc} 0.3221 & 0.0000\\ 0.0000 & 0.3220 \end{array}\right]$

The combination of discount factor (δ) and V that optimized the models for each training set is shown in Table 3.

**Table 3:** Values for the discount factor (*δ*) and observational variance (V) obtained from the different learning sets with mortality and antibiotic consumption data for weaner, sow and finisher herds.

Data stream	Herd	Discount factor (*δ*)	Observational variance (*V*)
Mortality	Weaner	0.90	0.00096
	Sow	1.00	0.00002
	Finisher	0.99	0.00092
Total antibiotic consumption	Weaner	0.86	5.77
	Sow	0.90	0.04
	Finisher	0.88	0.19
Antibiotic consumption for respiratory diseases	Weaner	0.51	0.27
	Sow	0.94	0.04
	Finisher	-	-
Antibiotic consumption for reproductive and urogenital diseases	Sow	0.74	1.91

The burn-in period of the DLMs was found to be 10 observations (months) for all herds.

Based on visual inspection of the MAD between the optimized and randomly initiated DLMs (presented in S2 Appendix), we find that the DLMs are stable against variations to the initial prior distribution, once the burn-in period of the models (10 observations) has been completed.

### Model performance

The specificities of the different monitoring methods, when applied to the healthy test sets are presented in Table 4. The block sensitivities of the different monitoring methods, when applied to the non-healthy test sets, are presented in Table 5. The best Youden J index values found for each age group, along with the respective methods used to achieve them, are presented in Table 6.

**Table 4:** Specificity for the different monitoring methods when applied to different healthy test sets.

	Monitoring method (N)	DLM forecast errors		DLM growth		Smoothed growth	
		2σ	3σ	95% CI^d^	99% CI^d^	95% CI^d^	99% CI^d^
Weaner	Mortality (4454)	0.98	0.98	0.97	0.98	0.96	0.97
	Ab total^a^ ‘(1380)	0.99	0.99	0.99	0.99	0.99	0.99
	Ab resp^b^ (184)	0.76	0.77	0.73	0.74	0.93	0.93
Sow	Mortality (4600)	0.94	0.96	0.92	0.95	0.72	0.77
	Ab total^a^ (1932)	0.96	0.98	0.94	0.97	0.88	0.92
	Ab resp^b^ (690)	0.76	0.77	0.76	0.77	0.91	0.92
	Ab repro^c^ (1840)	0.93	0.95	0.91	0.93	0.90	0.93
Finisher	Mortality (4462)	0.88	0.89	0.87	0.88	0.93	0.95
	Ab total^a^ (1012)	0.93	0.94	0.92	0.93	0.93	0.95

^a^Ab total: Total antibiotic consumption

^b^Ab resp: Antibiotic consumption for respiratory diseases

^c^Ab repro: Antibiotic consumption for reproductive and urogenital diseases

^d^CI: Credible intervals

N: number of observations included (calculated based on the number of herds multiplied by the number of months (46 months) included in the training sets)

DLM: Dynamic linear model

2σ: 2 standard deviations

3σ: 3 standard deviations

**Table 5:** Results for the block sensitivity achieved for each monitoring method, calculated for blocks of time from 4 months prior to 4 months posterior to herds becoming positive for a given disease.

		Porcine reproductive and respiratory syndrome positive herds						Porcine Pleuropneumonia positive herds					
		DLM forecast errors		DLM growth		Smoothed growth		DLM forecast errors		DLM growth		Smoothed growth	
		2σ	3σ	95% CI^d^	99% CI^d^	95% CI^d^	99% CI^d^	2σ	3σ	95% CI^d^	99% CI^d^	95% CI^d^	99% CI^d^
Weaner	Mortality	0.00	0.00	0.00	0.00	0.00	0.00	0.14	0.14	0.00	0.00	0.00	0.00
	Ab total^a^	0.05	0.00	0.00	0.00	0.00	0.00	0.05	0.05	0.00	0.00	0.00	0.00
	Ab resp^b^	0.11	0.00	0.22	0.11	0.22	0.22	0.00	0.00	0.00	0.00	0.00	0.00
Sow	Mortality	0.27	0.03	0.17	0.03	0.30	0.30	0.25	0.15	0.20	0.05	0.30	0.30
	Ab total^a^	0.12	0.08	0.38	0.33	0.38	0.38	0.00	0.00	0.25	0.25	0.30	0.25
	Ab resp^b^	0.09	0.09	0.00	0.00	0.09	0.09	0.25	0.17	0.50	0.50	0.50	0.50
	Ab repro^c^	0.00	0.00	0.00	0.00	0.00	0.00	-	-	-	-	-	-
Finisher	Mortality	0.03	0.00	0.08	0.03	0.22	0.25	0.00	0.00	0.45	0.45	0.30	0.45
	Ab total^a^	0.12	0.00	0.41	0.24	0.47	0.47	0.23	0.23	0.23	0.00	0.38	0.38
	Ab resp^b^	0.00	0.00	0.00	0.00	0.00	0.00	0.00	0.00	0.00	0.00	0.00	0.00

^a^Ab total: Total antibiotic consumption

^b^Ab resp: Antibiotic consumption for respiratory diseases

^c^Ab repro: Antibiotic consumption for reproductive and urogenital diseases

^d^CI: Credible intervals

DLM: Dynamic linear model

2σ: 2 standard deviations

3σ: 3 standard deviations

**Table 6:** Summary of the best predictive performances achieved for each age group, as measured by Youden’s J index. Also shown are the methods, specificities, and block sensitivities associated the best J indexes for each age group.

Disease	Age group	Best J index	Best data to monitor	Best monitoring method	Specificity	Block sensitivity
PRRS^a^	Weaner	0.49	Ab total^c^	DLM, forecast error, 2σ	0.99	0.50
	Sow	0.82	Ab resp^d^	Smoothed growth, 99% CI	0.92	0.90
	Finisher	0.67	Mortality	DLM, growth, 99% CI	0.87	0.80
PP^b^	Weaner	0.49	Ab total^c^	DLM, forecast error, 3σ	0.99	0.50
	Sow	0.42	Ab resp^d^	Smoothed growth, 99% CI	0.92	0.50
	Finisher	0.33	Mortality	Smoothed growth, 99% CI	0.95	0.38

^a^ PRRS: Porcine reproductive and respiratory syndrome

^b^ PP: Porcine Pleuropneumonia

^c^ Ab total: Total antibiotic consumption

^d^Ab resp: Antibiotic consumption for respiratory diseases

The percentage of herds with alarms based on mortality data increased by 3% for sows up to 2 months before they became PRRS-positive based on 2σ and 95% CI for the growth component (Fig A in S3 Appendix). Looking at the total antibiotic consumption, an increasing shift (≥11%) occurred for 1 month prior for finisher herds and 3 months prior for sow herds based on the growth component and the smoother (Fig B in S3 Appendix). In terms of antibiotic consumption for respiratory diseases, 11% of weaner herds had alarms 2 months prior (Fig C in S3 Appendix–using CI on the smoother), while sow herds had alarms 1 month after they shifted to a PRRS-positive status (based on Shewhart control chart based monitoring methods and the smoother).

For weaner, sow, and finisher herds that became porcine pleuropneumonia-positive, increases (>5%) in the percentage of alarms were seen between 3 months prior and 2 months after based on the different methods for mortality based on Shewhart control chart based monitoring methods and growth component of the DLM (Fig D in S3 Appendix). The highest percentages of alarms were achieved (≥ 10%) for sows and finisher herds up 2 months prior to serological confirmation, based on the growth component obtained from the smoother. Peaks in the percentage of weaner herds with alarms based on mortality were seen every 2 months based on Shewhart control chart based monitoring methods (Fig D in S3 Appendix). In terms of total antibiotic consumption, ≥ 5% of weaner and finisher herds generated alarms in the same month (month 0) that they became porcine pleuropneumonia-positive based on σ (Fig E in S3 Appendix). Interestingly, peaks in the percentage of finisher herds with alarms were seen 3 months apart based on σ (Fig E in S3 Appendix). The highest increases in the % of herds with alarms observed for sows and finishers was ≥ 16% and it happened 3 months before and in month 0 respectively based on the growth component obtained from the smoother. Based on the six monitoring methods, the percentage of sow herds with alarms based on antibiotic consumption for respiratory diseases increased from 0% to 8% in the 1 month prior to these herds becoming porcine pleuropneumonia-positive (Fig F in S3 Appendix).

The methods that generated the highest percentage of herds with alarms were based on monitoring changes in growth both from the DLM and from the smoother.

Shifts based on antibiotic consumption for respiratory diseases were observed to be higher for porcine pleuropneumonia when compared to herds that became PRRS-positive and compared to alarms based on mortality and total antibiotic prescription.

## Discussion

Changes in mortality data and antibiotic consumption at herd level were monitored in Danish swine herds that became positive for Porcine Reproductive and Respiratory Syndrome (PRRS) or porcine pleuropneumonia. These diseases were included in this study because they are endemic in Denmark and are part of a voluntary surveillance system, allowing us to follow up on herds on a regular basis. Mortality and antibiotic consumption data were included in the study because they are indicators of outbreaks and are available for all Danish farms at herd level, thus making them good potential proxies for national disease monitoring efforts.

### Study design

We chose to monitor: 1) changes in antibiotic consumption for respiratory diseases because both PRRS in combination with other etiology and *Actinobacillus pleuropneumoniae* cause clinical pneumonia [45]; 2) data on antibiotic consumption for reproductive and urogenital diseases from sow herds due to the symptoms caused by PRRS [45]; 3) the total consumption of antibiotics due to possible misclassification of the disease groups registered in VetStat and because antibiotics prescribed for other groups of diseases (such as gastrointestinal or systemic diseases) may be used to treat respiratory diseases.

Changes in the percentage of herds generating alarms were analyzed from 4 months prior to 4 months after serologically observed changes in the disease status, because we assumed that subclinical and chronic cases would occur within this period, following the example of [46]. Moreover, the frequency of serological tests also depends on the herd type, varying from intervals of 4 weeks [47] up to 12 months, and seroconversion requires a minimum timespan (1–2 weeks for the IPMA test for PRRS [48]).

Due to the availability of control measures such as vaccination or health management programs, the dynamics of disease spread are expected to be different for endemic diseases, resulting in a lower incidence rate compared to exotic diseases. Moreover, the natural immunity developed from previous exposure to the disease agent also reduces an animal’s susceptibility (i.e. causing mild clinical symptoms) and contributes to the maintenance of the infection in the herds. The same farm can have both healthy and sick animals in different herds. In cases of outbreaks within a farm, it is expected that farmers will take measures to avoid the spread of disease, and that they thus will continue to also have disease-negative herds. One possible management solution is total or partial depopulation-repopulation; in this case, the number of infected animals would be reduced, resulting in lower mortality rates and/or antibiotic consumption. These issues may explain the relatively low percentage of herds with alarms based on changes in mortality and antibiotic consumption (S3 Appendix).

### Findings

In this study, we estimated the overall mortality and antibiotic usage for the various age groups in Danish pig production. These estimates were used as initial prior values for our time series modeling. Unfortunately, comparing these priors to superficially similar values in the published literature is not meaningful; pig breeds, herd management, and national disease surveillance strategies vary greatly between countries, which influence the morbidity of PRRS and porcine pleuropneumonia. Additionally, the mortality data used in this study was provided by the Danish Veterinary and Food Administration and they are calculated based on the SMD and the CHR. These data have not been used to quantify the mortality at herd level for healthy Danish swine herds in previous studies. Regarding the antibiotic consumption data, previous studies use data at purchase level, which is not comparable to our data at monthly scale at herd level.

Our findings suggest that increases on antibiotic consumption and mortality can be found in Danish swine herds prior to, during and after the confirmation of a positive disease status based on serological diagnostic test results. These findings are in concordance with previous studies in which increases of antibiotic usage and mortality were found prior and posterior to the time when swine herds got infected with other infectious diseases, such as post weaning multisystemic wasting syndrome and enzootic pneumonia [18, 19, 46].

The absence of weaner herds becoming PRRS-positive with alarms based on mortality data (Fig A in S3 Appendix) might be explained by the use of systematic metaphylactic treatments (prophylactic treatment against a previously diagnosed disease within the herd), mainly used for gastrointestinal diseases [25]. This practice would protect weaners against secondary respiratory infections and, as a consequence, lower the mortality caused by the PRRS virus, thus leading to an absence of alarms in these herds. Moreover, the registration of dead weaners based on the number of containers transported from a farm to the rendering plant could result in a bias. Mortality estimations use a fixed number of dead weaners per container. This can lead to incorrect calculations–most likely an underreporting of dead weaners, due to the fact that farmers must pay for the transport of these containers to the rendering plant. An increase in the percentage of herds with alarms based on mortality was seen for the three herds that got a porcine pleuropneumonia positive status (Fig D in S3 Appendix and Table 5) due to the fact that this disease causes high mortality [45]. This disease also causes severe respiratory distress, dyspnea, and a persistent cough, especially in young finishers [45], which necessitates the use of antibiotics to treat secondary infections and reduce symptoms (as seen in Figs E and F in S3 Appendix).

When considering antibiotic consumption, it is important to note that the recorded date in the VetStat database is the date of purchase and not the date of treatment. According to the legislation, veterinarians may prescribe antibiotics for diseases expected to arise in the period before the next veterinary consultation (typically 1 month). In this study, we assumed that all antibiotics purchased were used at a constant rate between two consecutive purchases or within 90 days. This might represent a limitation, as it does not take into account the duration of treatments, fluctuation in medicine prices, and variation among veterinarians, and may result in the observed “peaks” in the percentage of herds with alarms every 2–3 months seen for antibiotic consumption in herds becoming Porcine pleuropneumonia-positive (Fig C in S3 Appendix). Interestingly, increases in the percentage of herds that became porcine pleuropneumonia-positive with alarms based on antibiotic consumption for respiratory diseases were only seen for sows (Fig F in S3 Appendix). A more constant use of antibiotics for the metaphylaxis of gastrointestinal diseases, particularly in weaners and in some young finishers [25], may cause fewer respiratory symptoms in porcine pleuropneumonia-positive herds with chronic infections. And when an outbreak occurs, antibiotics prescribed for gastrointestinal disease are likely already present on the farm and may be used for treatment of respiratory disease. This may explain why the antibiotic prescription for respiratory diseases in case of outbreaks is lower when compared to sow herds (Fig F in S3 Appendix). This is also the reason for the low specificity consistently seen when the DLM was applied to the use of antibiotics for respiratory diseases (Table 4); essentially, the baseline use of these specific antibiotics is so low that almost any use at all will result in an alarm. In general, the percentage of non-healthy herds with alarms was higher when monitoring changes in the growth component obtained from either the DLM or from the smoother (i.e. retrospective analysis). As an example, this is evident when looking at antibiotic consumption for respiratory diseases in newly PRRS-positive herds (Fig C in S3 Appendix), where >10% of weaner herds had alarms prior to the change in the disease status. When using the smoother, both observed and filtered data obtained from the DLM data are used to make an estimate, increasing the level of confidence in the smoother mean and growth, which will result in narrower CI and consequently improved ability to detect changes. One noticeable exception to this, however, is the weaner herds, where the DLM-forecast error-based alarms are consistently the best, as seen from Tables 5 and 6. This is explained by impact of the diseases be more severe in weaners when compared to sows and finishers [24].

When looking at which data had the highest J index (Table 6), the mortality data proofed to be the best data to monitor when looking at outbreak in finishers. This might be explained by the fact that young finishers have high mortality due to disease, thermal stress, and nutrition [21] and that these herds have a lower antibiotic consumption when compared to weaners [25]. For sow herds, monitoring changes on the antibiotic consumption for respiratory diseases seemed to be the best approach to detect outbreaks of PRRS and porcine pleuropneumonia due to the lower general consumption of antibiotics group and very low use of systematic metaphylactic treatments (prophylactic treatment against a previously diagnosed disease within the herd) in these herds. For weaners, the best data to monitor was the total of antibiotic usage possible because these animals have more severe symptoms (when compared to finishers and sows) due to the presence of diseases, thermal stress, and nutrition [24], which leads the farmers to use antibiotics more frequently in case of outbreaks.

The results showed that no single method or type of data are universally “the best” at changes from negative to positive herds; there were differences in the number of herds generating alarms occur to on the age group, the disease, the data and the monitoring method used. This was likely due to the factors explained in the previous paragraphs. Therefore, the choice of which data to monitor and the temporal monitoring method would have to be made individually for each age group and disease. The decision to take actions should be taken after combining the information provided by these statistical approaches, the epidemiological situation and the economic impact of the (re-)emerging and endemic diseases. Also, the extent to which these data can be used as indicators of swine diseases varies; these data contain information, which can be used in different steps of disease monitoring and surveillance, including 1) data monitoring, 2) defining control and preventive measures, and 3) follow-up.

### Perspectives

Further epidemiological research will be needed to evaluate different approaches for monitoring changes in the data that could indicate spread of disease. This depends on the targeted disease (emerging vs endemic) and on the choice of the study unit of disease monitoring and surveillance (herd, regional, or national level). The use of these methods based on the different data streams in parallel ensures that the chances of detection increase for different outbreak scenarios. To our knowledge, this approach has not previously been applied in veterinary sciences. Methods to implement dynamic linear models for monitoring changes in trends jointly using multiple data streams in parallel will be evaluated in future steps of this work. Improving data quality for disease monitoring and surveillance requires that the identified limitations are addressed. The development of mobile phone applications to update the number of animals within the herds could be worth considering to improve the accuracy of the CHR, which is used to calculate the mortality at farm level. Good and updated data on herd size is crucial for the calculations of mortality. Specifically for the Danish data, the recording of number of dead weaners also needs improving. For disease surveillance purposes, the data registered in VetStat should include the date of treatment instead of (or as a supplement to) the date of prescription, requiring direct reports from farmers to VetStat, thus enabling by new IT solutions. This would allow us to improve the quality of the data used with the potential to better monitor changes in consumption of antibiotic usage.

## Conclusions

Monitoring changes in mortality data and antibiotic consumption (in total and for treatment of respiratory diseases) showed changes (i.e. alarms) at herd level prior to diagnostic test confirmation. These results also show a potential value for using these data streams as a proxy for outbreaks when monitoring diseases as part of surveillance strategies. What data type is most informative for this purpose differs consistently between the three age groups due to differences in typical management and data reporting for these different age groups.

## Supporting information

S1 Appendix R code. (PDF)

S2 Appendix Assessments of the computational stability given the initial prior distributions. This was assessed by repeatedly (N = 20) initiating the DLM with a randomly chosen initial prior distribution. Subsequently, the mean absolute deviations (MAD) between the filtered means of the optimized models and the filtered means from the models initiated with randomly chosen distributions were calculated for each time step. The red line (t = 10) represent the burn in period of the models in the different data sets.
(PDF)

S3 Appendix Percentage of weaner, sow, and finisher herds with alarms based on mortality and antibiotic consumption data obtained prior to, during, and after becoming positive for Porcine Reproductive and Respiratory Syndrome (PRRS) or Porcine pleuropneumonia (caused by *Actinobacillus pleuropneumoniae*) (month = 0). The alarms were generated based on Shewhart control charts (using 2σ and 3σ thresholds) applied to standardized forecast errors, and on the growth component (based on 95% CI and 99% CI) obtained from a Dynamic Linear Model (DLM) with a Kalman filter performing prospective analysis, as well as the growth component (based on 95% CI and 99% CI) obtained from a smoother performing retrospective analysis. The number of herds included in these calculations can be found in Table 1.
Fig A. Percentage of weaner, sow, and finisher herds with alarms based on mortality data obtained prior to, during, and after becoming positive for Porcine Reproductive and Respiratory Syndrome (PRRS) (month = 0).
Fig B. Percentage of weaner, sow, and finisher herds with alarms based on the total antibiotic consumption data obtained prior to, during, and after becoming positive for Porcine Reproductive and Respiratory Syndrome (PRRS) (month = 0).
Fig C. Percentage of weaner, sow, and finisher herds with alarms based on antibiotic consumption for respiratory diseases data obtained prior to, during, and after becoming positive for Porcine Reproductive and Respiratory Syndrome (PRRS) (month = 0).
Fig D. Percentage of weaner, sow, and finisher herds with alarms based on mortality data obtained prior to, during, and after becoming positive for Porcine pleuropneumonia (caused by *Actinobacillus pleuropneumoniae*) (month = 0).
Fig E. Percentage of weaner, sow, and finisher herds with alarms based on the total antibiotic consumption data obtained prior to, during, and after becoming positive for Porcine pleuropneumonia (caused by *Actinobacillus pleuropneumoniae*) (month = 0).
Fig F. Percentage of weaner, sow, and finisher herds with alarms based on antibiotic consumption for respiratory diseases data obtained prior to, during, and after becoming positive for Porcine pleuropneumonia (caused by *Actinobacillus pleuropneumoniae*) (month = 0).
(PDF)
