# Supplementary material for: Unweaving tangled mortality and antibiotic consumption data to detect disease outbreaks – Peaks, growths, and foresight in swine production
Source: PLoS One. 2019 Oct 9;14(10):e0223250. doi: 10.1371/journal.pone.0223250 (PMC6785175; doi:10.1371/journal.pone.0223250)
Supplement: S1 Appendix — (PDF) [file pone.0223250.s001.pdf]

```
##### DLM FUNCTIONS
f.uni <- matrix(c(1,0),nrow=2)
G.uni <- matrix(c(1,1,0,1),byrow=T,nrow=2)

make.matrix.symmetrical <- function(MATRIX){
  DIMS <- dim(MATRIX)
  new.matrix <- matrix(rep(NA, DIMS[1]*DIMS[2]), byrow = TRUE, nrow = DIMS[1])
  if(DIMS[1] == DIMS[2]){
    for(i in 1:DIMS[1]){
      for(j in 1:DIMS[1]){
        value.a <- MATRIX[i,j]
        value.b <- t(MATRIX)[i,j]
        value.mean <- mean(c(value.a, value.b))
        new.matrix[i,j] <- value.mean
      }
    }
  }else{
    print('WRONG!')
  }
  return(new.matrix)
}


alg84.dlm = function(k,f=f.uni,G=G.uni,V=NA,delta,N, C0=C0, mu0=mu0) {

  ##store results
  e <- rep(NA,N)
  et.norm <- rep(NA,N)
  et.abs <- rep(NA,N)
  pred <- rep(NA,N)
  Q <- rep(NA,N)
  growth <- rep(NA,N)
```

```
growth.var <- rep(NA,N)
M.filtered <- list()
C <- list()
a <- list()
R <- list()
A <- list()

prev <- na.omit(unlist(k))
mt2 <- mu0
Ct2 <- C0



# Iterate over observations
for(t in 1:N){

  if(prev[t]==0){

    mt <- at
    Ct <- Rt
    et <- NA
    Qt <- NA
    error.norm  <- NA

  } else{

    # Prior mean
    at <- G %*% mt2
                  # Prior Variance for theta
    Pt=G %*% Ct2 %*% t(G)
    Rt=(1/delta) * Pt
    Rt=make.matrix.symmetrical(Rt)
```

```
  # One-step Forecast mean
  ft <- t(f) %*% at
  # One-step Forecast variance
  if(is.na(V)){
    Qt <- t(f) %*% Rt %*% f + (((prev[t])*(1-(prev[t]))))
  }else{
    Qt <- t(f) %*% Rt %*% f + V
  }



  # Adaptative Coef. matrix
  At <- (Rt %*% f) %*% solve(Qt)

  #Get forecast error and MSE
  et <- prev[t]- ft
  error.norm <- et/sqrt(abs(diag(Qt)))

  ##updating equations
  mt <- at + At %*% et
  Ct <- Rt - (At %*% Qt %*% t(At))
  Ct <- make.matrix.symmetrical(Ct)

}

##start all over again:make them prior
mt2 = mt
Ct2 = Ct

# store values
C[[t]] <- Ct
a[[t]] <- at
```

```
    R[[t]] <- Rt
    # store prediction and error
    pred[t] <- ft
    e[t] <- et
    et.norm[t]<-error.norm
    A[[t]] <- At

    Q[t] <- Qt
    #if(length(mt)==2){
      growth[t] <- mt[2,1]
    #}

    M.filtered[[t]] <- mt
    # get growth variance..
     f.growth <- matrix(c(0,1),nrow=2)
     growth.var[t] <- (t(f.growth)%*%Ct)%*%f.growth



  }



  return(list(f=pred,e=e,et.norm=et.norm,q=Q,growth=growth,
              growth.var = growth.var, M.filtered=M.filtered,
              C = C, a = a, R = R, A = A))
}



###### SMOOTHER FUNCTIONS
```

```
Gt <- G.uni

runSmoother <- function(res) {

  n = length(res$M.filtered)
  mts <- list();
  Cts <- list();

  # Put last value equal to filtered
  mts[[n]] <- res$M.filtered[[n]]
  Cts[[n]] <- res$C[[n]]

  # These are useful
  Bt <- list()
  Lt <- list();
  growth.var <- list()
  growth.var[[n]] <- res$growth.var[[n]]

  # Iterate backwards over days
  for(i in ((n-1):1))   {

    #print(i)
    res$R[[i+1]] <- as.matrix(res$R[[i+1]])

    Bt[[i]] <- as.matrix( res$C[[i]] %*% t(Gt) %*% solve(res$R[[i+1]]) )
    mts[[i]] <- res$M.filtered[[i]] + Bt[[i]] %*% (mts[[i + 1]] - res$a[[i+1]])
    Cts[[i]] <- as.matrix( res$C[[i]] + Bt[[i]] %*% (Cts[[i+1]] - res$R[[i+1]]) %*% t(Bt[[i]]) )

    f.growth <- matrix(c(0,1),nrow=2)
    growth.var[[i]] <- (t(f.growth)%*%Cts[[i]])%*%f.growth

  }
```

```
  return(list(mts=mts,
              Cts=Cts,
              Lt=Lt,
              growth.var=growth.var));
}


###### OPTIMIZE DELTA AND V

get.delta.v.mort <- function(data.set, N,
                             deltamin, deltamax, deltainc,
                             Vsmin, Vsmax, Vsinc){
  relevant.data <- as.vector(as.matrix(data.set[,1:N]))
  relevant.data.set <- data.set[,1:N]
  m0 <- mean(na.omit(relevant.data))
  mu0 <- as.matrix(c(m0,0),ncol=1)
  C0 <- diag(c(m0/10, m0/100))
  N <- N
  Vs <- c(NA, seq(Vsmin, Vsmax, Vsinc))
  Vs <- Vs

  out.all <- cbind()
  for(V in Vs){
    print('############################')
    print(paste('V = ', V))
    best.delta <- getdelta4(data.set, delta.max=deltamax, delta.min=deltamin, incre=deltainc,
                            N=N, C0=C0, mu0=mu0,
                            silent=FALSE, V=V)
    errors.out <- look.at.errors4(data.set, best.delta, N, V=V)
    a <- errors.out$a
```

```
    MAE <- round(errors.out$MAE, digit=2)

    out <- cbind('V'=V, 'Best.Delta'=best.delta, 'a'=a, 'MAE'=MAE)
    out.all <- rbind(out.all, out)
  }
  out.all <- as.data.frame(out.all)
  print(out.all)}



###### RUN DLM AND SMOOTHER
run.dlm.smoother <- function(data.set, delta, V, N){

  out.all <- list()
  relevant.data <- as.vector(as.matrix(data.set[,1:N]))
  relevant.data.set <- data.set[,1:N]
  m0 <- mean(na.omit(relevant.data))
  mu0 <- as.matrix(c(m0,0),ncol=1)
  C0 <- diag(c(m0/10, m0/100))

  for (i in 1:dim(data.set)[1]){
      print(i)

    ## run dlm and smoother
    res.dlm <- alg84.dlm.new( k = as.numeric(relevant.data.set[i,]),
                          f = f.uni, G = G.uni, delta = delta,
                          N = dim(relevant.data.set)[2],
                          V = V, C0 = C0, mu0 = mu0)

    et.norm <- res.dlm$et.norm
    growth  <- res.dlm$growth
    growth.var  <- res.dlm$growth.var
```

```
M.filtered <- unlist(res.dlm$M.filtered)[seq(from=1, to=length(unlist(res.dlm$M.filtered)), by=2)]

G.filtered <- unlist(res.dlm$M.filtered)[seq(from=2, to=length(unlist(res.dlm$M.filtered)), by=2)]

C.level <- unlist(lapply(res.dlm$C, diag))[seq(from=1, to=length(unlist(res.dlm$M.filtered)), by=2)]

C.growth <- unlist(lapply(res.dlm$C, diag))[seq(from=2, to=length(unlist(res.dlm$M.filtered)), by=2)]

A.m <- unlist(res.dlm$A)[seq(from=1, to=length(unlist(res.dlm$A)), by=1)]

A.g <- unlist(res.dlm$A)[seq(from=2, to=length(unlist(res.dlm$A)), by=2)]



res.smooth <- runSmoother(res=res.dlm)


mts.mean <- unlist(res.smooth$mts)[seq(from=1, to=length(unlist(res.smooth$mts)), by=2)]

mts.growth <- unlist(res.smooth$mts)[seq(from=2, to=length(unlist(res.smooth$mts)), by=2)]

Cst.mean <- unlist(lapply(res.smooth$Cts, diag))[seq(from=1, to=length(unlist(res.smooth$Cts)),
                                                by=2)]

Cst.mean <- Cst.mean[!is.na(Cst.mean)]

Cst.growth <- unlist(lapply(res.smooth$Cts, diag))[seq(from=2, to=length(unlist(res.smooth$Cts)),
                                                  by=2)]

Cst.growth <- Cst.growth[!is.na(Cst.growth)]

Cst.growth.var <- unlist(res.smooth$growth.var)


## store results in tables

out.all.dlm <- cbind(et.norm, growth, growth.var)


out.all.smoother <- cbind(M.filtered, G.filtered, C.level,
                          C.growth, mts.mean,mts.growth, Cst.mean, Cst.growth, Cst.growth.var)
```

```
    out.all[[i]] <- cbind( out.all.dlm, out.all.smoother)

  }

  return(out.all)

}

#### calculate specificity
SP.table <- cbind()
for(name in unique(Test.set.healthy$Name)){
  print(name)

  Name.set <- subset(Test.set.healthy, Test.set.healthy$Name == name)
  # Get the number of observation in this sub-set
  N <- nrow(Name.set)*46
  # Get the DLM parameters
  nameA <- strsplit(name, 'healthy.')[[1]][2]
  delta <- best.params$Best.Delta[which(best.params$Name == name)]
  V <- best.params$V[which(best.params$Name == name)]
  mu0 <- get(paste('mu0.', nameA, sep=''))
  C0 <- get(paste('C0.', nameA, sep=''))

  # Run the DLM
  dlm.smo <- run.dlm.smoother.training.set2( data.set = Name.set[,1:46],
                                             delta = delta, V = V,
                                             mu0, C0)
```

```
  # Consider the smoothed values, and calculate their specificities
  smooth <- get.smoother.training(data.set = dlm.smo, ncol=46)
  SP.smooth <- unlist(sp.smoother.growth.training(data.input = smooth, ncol=46))

  ## calculate alarms based on DLM ----
  alarms <- alarms.testing.set(tst.results=dlm.smo)
  ## calculates specificity based on DLM ----
  sp.total <- unlist(tst.sp.total(data.set = alarms))

  # Combine specificites based on DLM and smoother
  sp.total <- c(N, round(c(sp.total, SP.smooth),2))
  sp.total <- c(name, sp.total)
  names(sp.total) <- c('Name', 'N',
                       'DLM.forecast_errors.2.SD', 'DLM.forecast_errors.3.SD',
                       'DLM.growth.95.%.CI', 'DLM.growth.99.%.CI',
                       'Smooth.growth.95.%.CI', 'Smooth.growth.99.%.CI')
  SP.table <- rbind(SP.table, sp.total)
}
SP.table <- as.data.frame(SP.table)




### get sensitivity
table.all <- cbind()
Se.all.all <- cbind()
for(name in unique(Test.set.sick$Name)){

  print(name)
  Name.set <- subset(Test.set.sick, Test.set.sick$Name == name)
```

```
#Get the number of herds - we'll need it for sensitivity calculations
N <- nrow(Name.set)

nameA <- strsplit(name, 'sick.')[[1]][2]
if(grepl(pattern = 'app.', x = nameA)){
  nameA <- strsplit(x = nameA, split = 'app.')[[1]][2]
}else{
  if(grepl(pattern = 'prrs.', x = nameA)){
    nameA <- strsplit(x = nameA, split = 'prrs.')[[1]][2]
  }
}

if(nameA == 'fin.resp'){
  nameA <- 'fin.adds'
}

# Add the word "healthy" just for looking up the values in the best.param table
nameB <- paste('healthy.', nameA, sep='')

# Get the DLM parameters
delta <- best.params$Best.Delta[which(best.params$Name == nameB)]
V <- best.params$V[which(best.params$Name == nameB)]
mu0 <- get(paste('mu0.', nameA, sep=''))
C0 <- get(paste('C0.', nameA, sep=''))

# Run the DLM
dlm.smo.test.set <- run.dlm.smoother.testing.set.finalversion(data.set = Name.set,
                                                              delta = delta,
                                                              V = V, B =-10)

alarms.out.test <- alarms.dlm.testing.set(tst.results=dlm.smo.test.set)
```

```
quant.test <- quantify.testing.alarmsV2(alarms.input = alarms.out.test,
                                        time.length=4)

# get smoother results and alarms
smo.out.test <- get.smoother.testing( data.set = dlm.smo.test.set,
                                      mincol = -46,
                                      maxcol = 46)

alarms.smo <- alarms.smoother.testing.set(tst.results = smo.out.test)

quant.test[[length(quant.test)+1]] <- alarms.smo[[1]]
quant.test[[length(quant.test)+1]] <- alarms.smo[[2]]

# Make a table out of it
methods <- c('DLM.forecast_errors.2.SD', 'DLM.forecast_errors.3.SD',
             'DLM.growth.95.%.CI', 'DLM.growth.99.%.CI',
             'Smooth.growth.95.%.CI', 'Smooth.growth.99.%.CI')
table.name <- cbind()
for(i in 1:length(quant.test)){
  a <- quant.test[[i]]
  table.name <- rbind(table.name,a)
}
table.name <- as.data.frame(table.name)
table.name$Method <- methods
table.name$Name <- name
table.name$N <- N

table.all <- rbind(table.all, table.name)
```

```
### Calculate block-sensitivity

methods.A <- c('DLM.forecast_errors.2.SD', 'DLM.forecast_errors.3.SD',
            'DLM.growth.95.%.CI', 'DLM.growth.99.%.CI', 'DLM.growth.pos2neg',
            'Smooth.growth.95.%.CI', 'Smooth.growth.99.%.CI')

Se.all <- c()
# - DLM based
alarms.out.test.A <- alarms.dlm.testing.set(tst.results=dlm.smo.test.set)
for(i in 1:length(alarms.out.test.A)){
   alarms.i <- alarms.out.test.A[[i]][,c('-4','-3','-2','-1','0','1','2','3','4')]
   col.sums <- c()
   for(j in 1:ncol(alarms.i)){
     col.sums <- c(col.sums,sum(na.omit(alarms.i[,j])))
   }
   Se <- length(which(col.sums>0))/N
   Se.all <- c(Se.all, Se)
}
# smoother based
alarms.smo.A <- alarms.smoother.testing.set.A(tst.results = smo.out.test)
for(i in 1:length(alarms.smo.A)){
  alarms.i <- alarms.smo.A[[i]][,c('-4','-3','-2','-1','0','1','2','3','4')]
  col.sums <- c()
  for(j in 1:ncol(alarms.i)){
    col.sums <- c(col.sums,sum(na.omit(alarms.i[,j])))
  }
  Se <- length(which(col.sums>0))/N
  Se.all <- c(Se.all, Se)
}

Se.all <- round(Se.all,2)
Se.all <- c(Se.all, name, N)
```

```
  names(Se.all) <- c(methods.A, 'Name', 'N')
  Se.all.all <- rbind(Se.all.all, Se.all)



}

table.all <- as.data.frame(table.all) ## time specific se

Se.all.all <- as.data.frame(Se.all.all)
for(i in c(1:length(methods.A),ncol(Se.all.all))){
  Se.all.all[,i] <- as.numeric(as.character(Se.all.all[,i]))
}
```
