# Supplementary figures and images for: Unweaving tangled mortality and antibiotic consumption data to detect disease outbreaks – Peaks, growths, and foresight in swine production
Source: PLoS One. 2019 Oct 9;14(10):e0223250. doi: 10.1371/journal.pone.0223250 (PMC6785175; doi:10.1371/journal.pone.0223250)

## Mean absolute deviation (MAD)

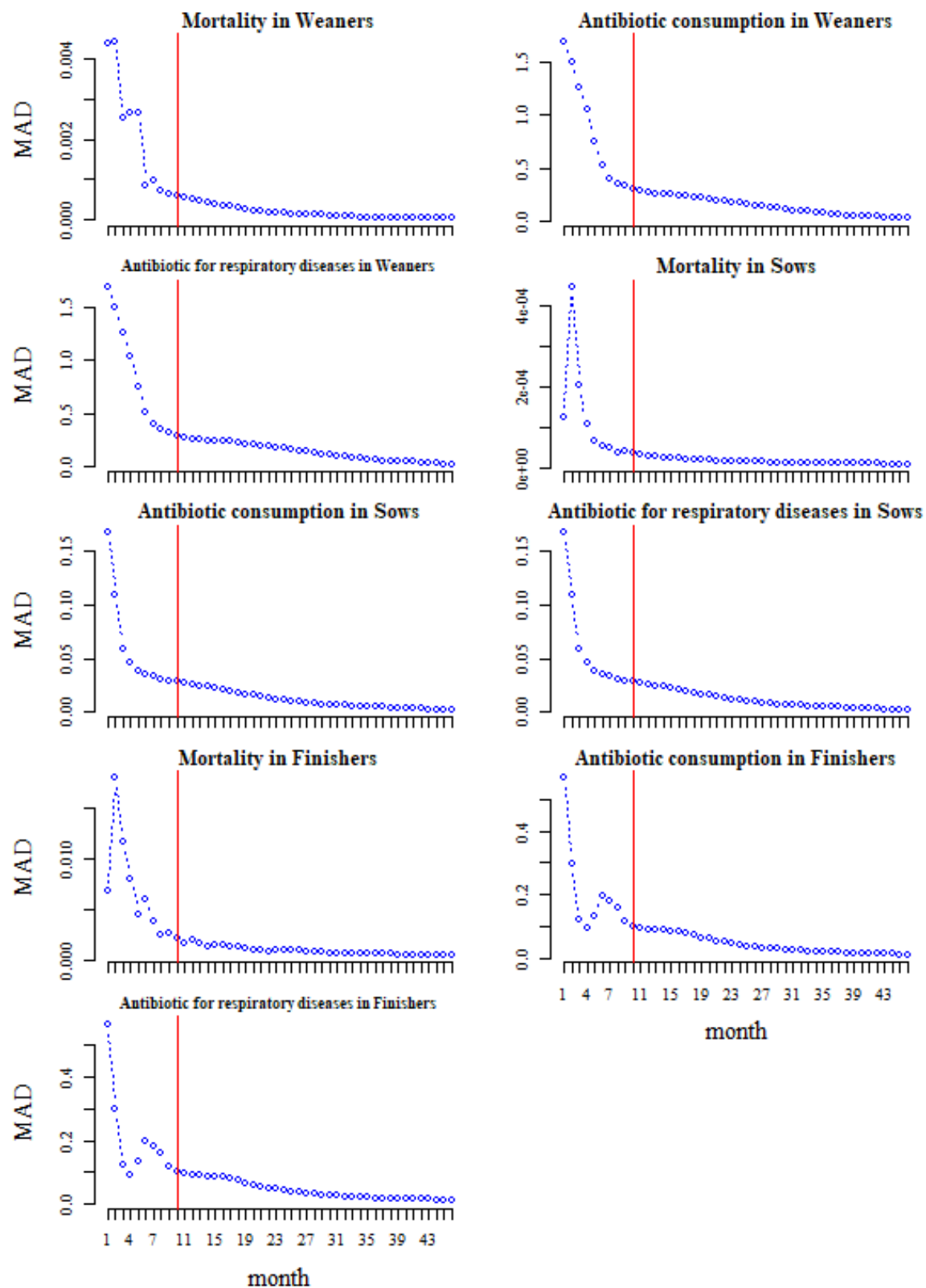

Supplement: S2 Appendix — This was assessed by repeatedly (N = 20) initiating the DLM with a randomly chosen initial prior distribution. Subsequently, the mean absolute deviations (MAD) between the filtered means of the optimized models and the filtered means from the models initiated with randomly chosen distributions were calculated for each time step. The red line (t = 10) represent the burn in period of the models in the different data sets. (PDF) [file pone.0223250.s002.pdf]
