# Supplementary material for: Unweaving tangled mortality and antibiotic consumption data to detect disease outbreaks – Peaks, growths, and foresight in swine production
Source: PLoS One. 2019 Oct 9;14(10):e0223250. doi: 10.1371/journal.pone.0223250 (PMC6785175; doi:10.1371/journal.pone.0223250)

Fig A. Percentage of weaner, sow, and finisher herds with alarms based on mortality data obtained prior to, during, and after becoming positive for Porcine Reproductive and Respiratory Syndrome (PRRS) (month=0).

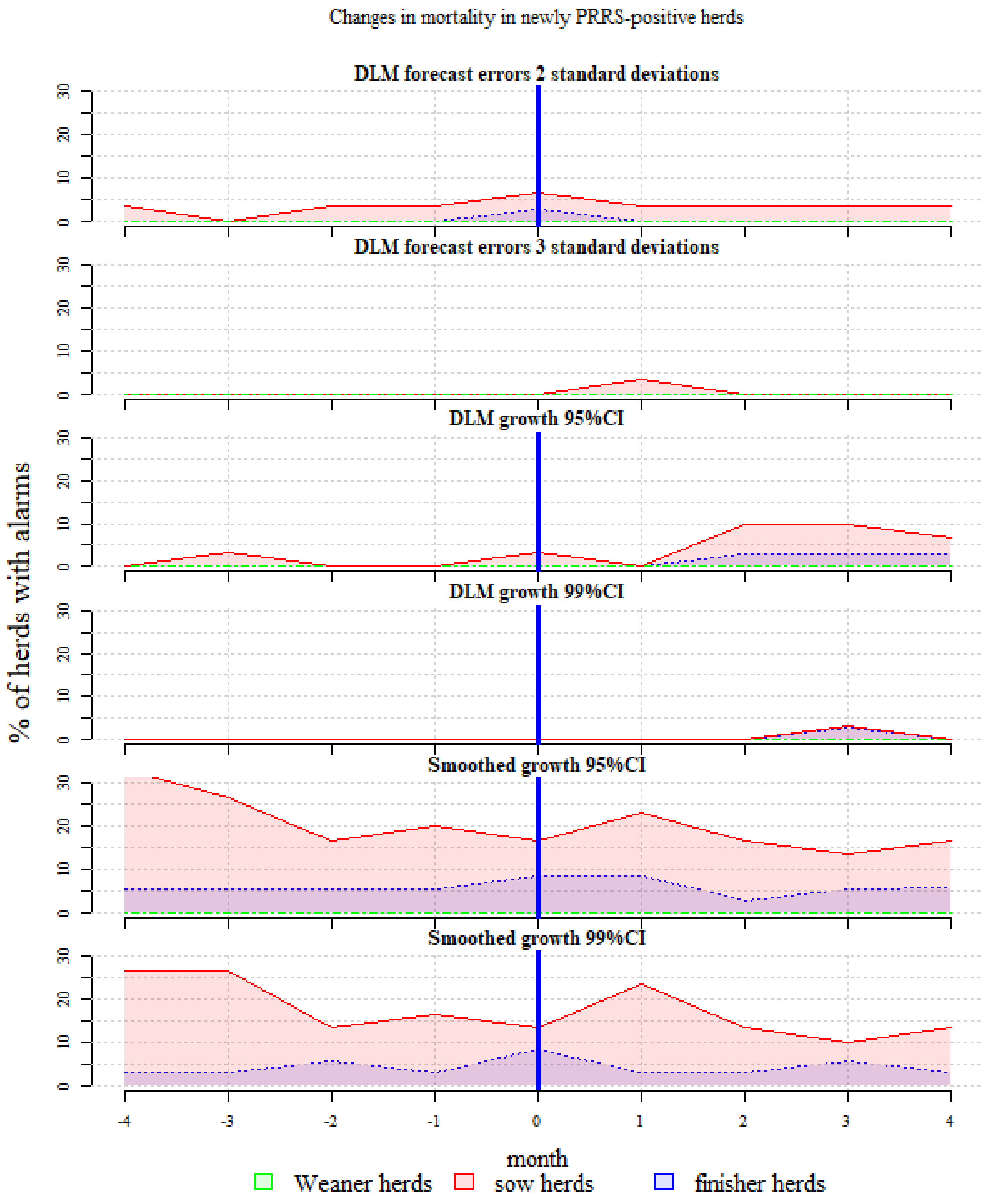

Fig B. Percentage of weaner, sow, and finisher herds with alarms based on the total antibiotic consumption data obtained prior to, during, and after becoming positive for Porcine Reproductive and Respiratory Syndrome (PRRS) (month=0).

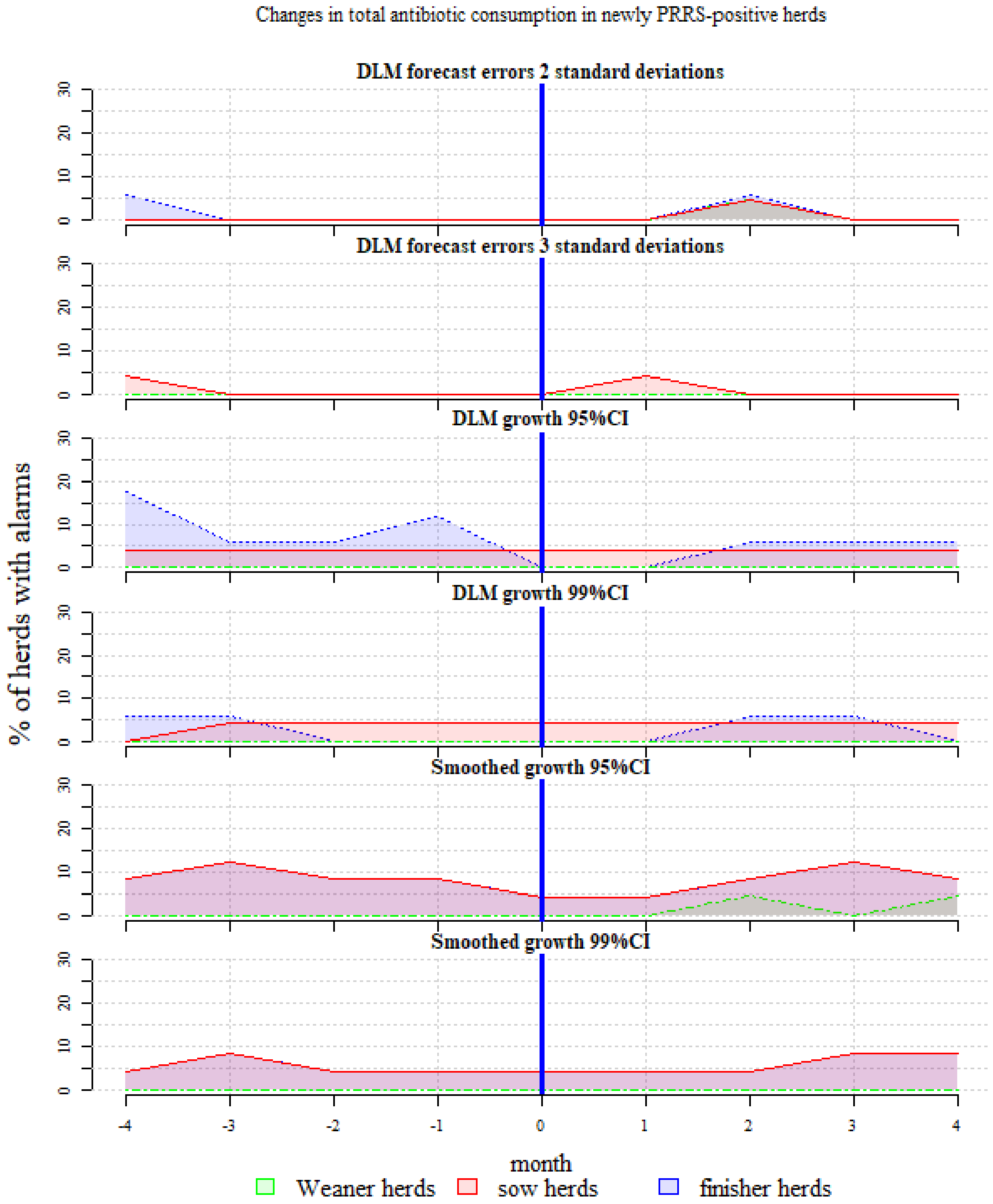

Fig C. Percentage of weaner, sow, and finisher herds with alarms based on antibiotic consumption for respiratory diseases data obtained prior to, during, and after becoming positive for Porcine Reproductive and Respiratory Syndrome (PRRS) (month=0).

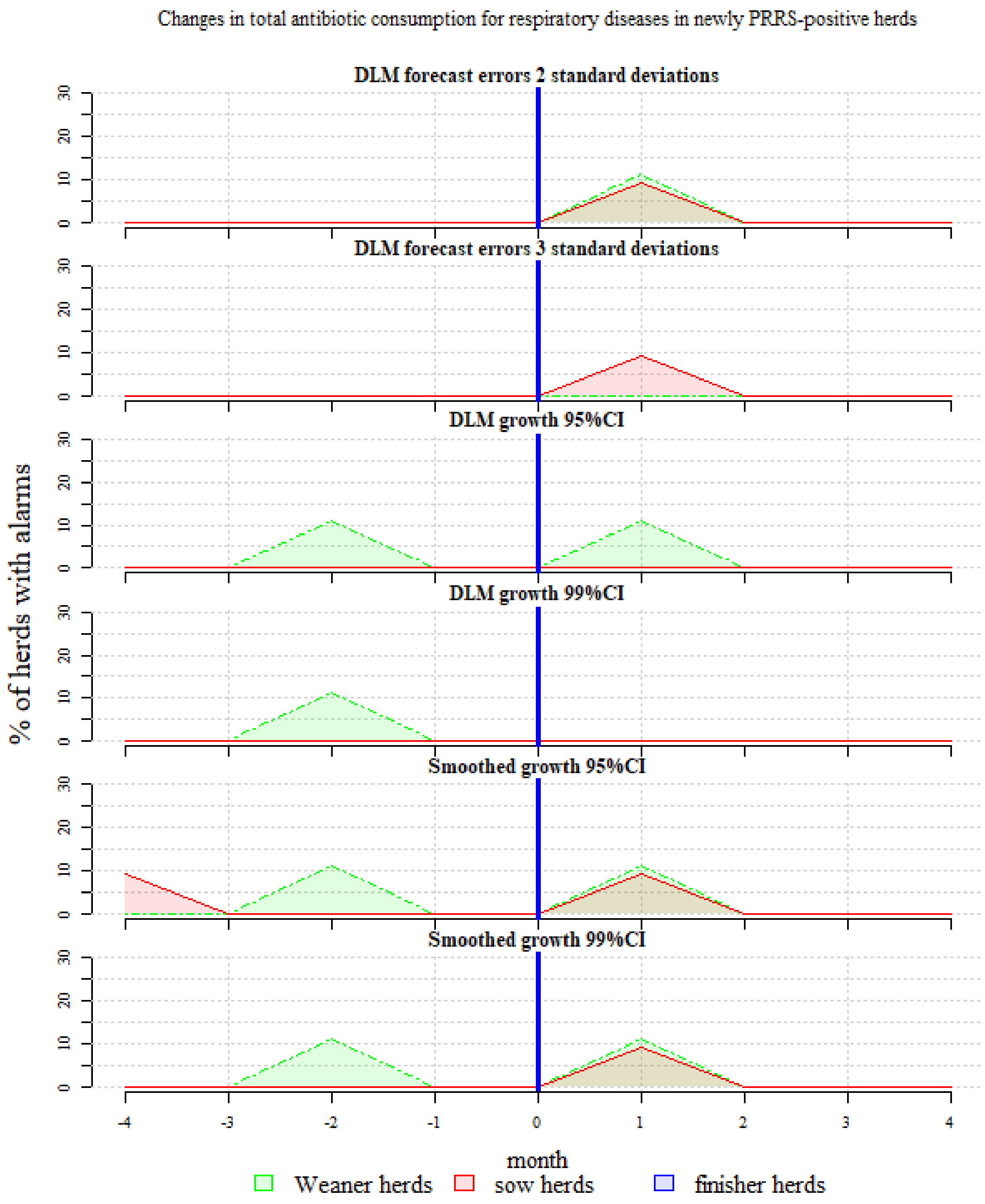

Fig D. Percentage of weaner, sow, and finisher herds with alarms based on mortality data obtained prior to, during, and after becoming positive for Porcine pleuropneumonia (caused by *Actinobacillus pleuropneumoniae*) (month=0).

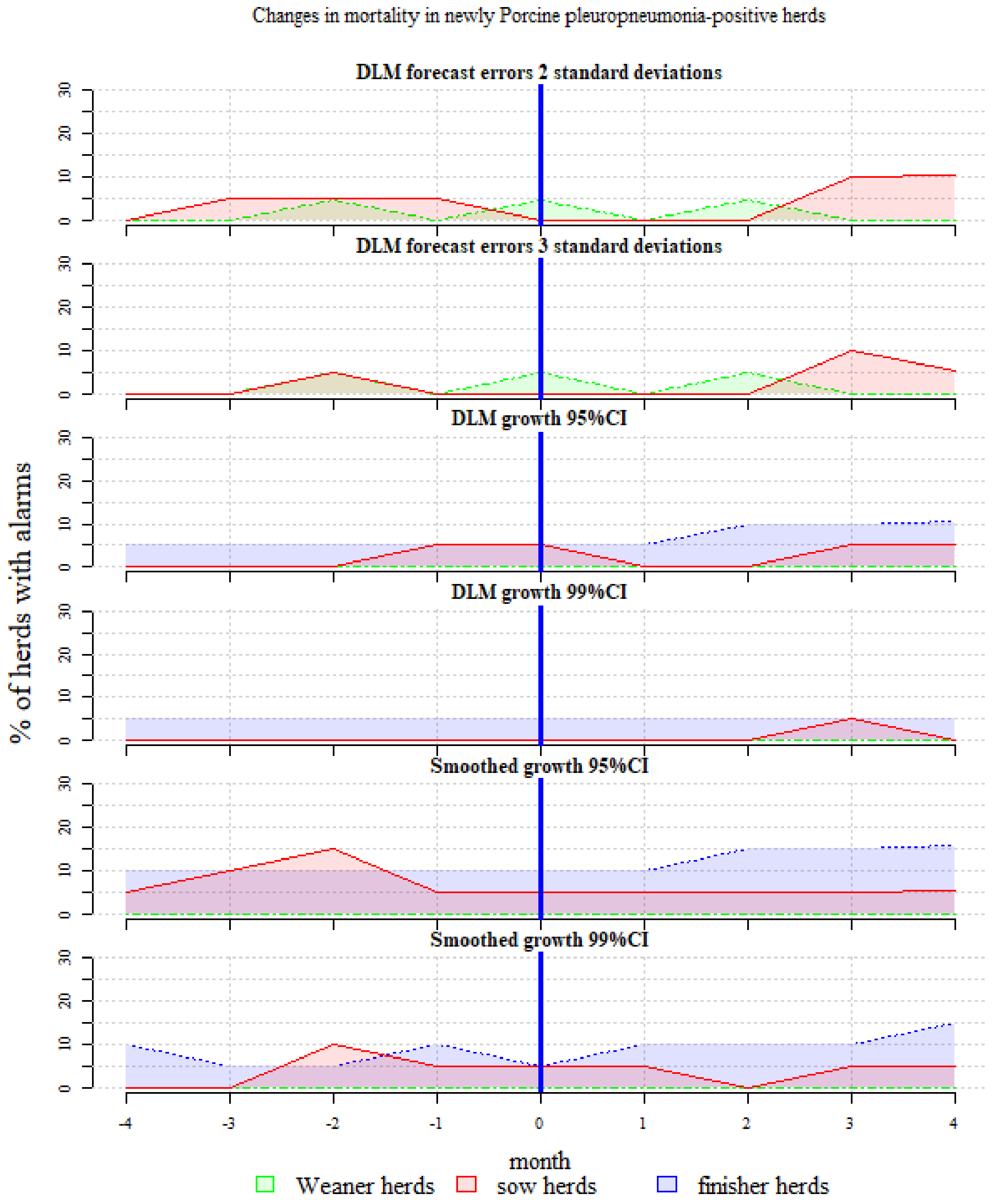

Fig E. Percentage of weaner, sow, and finisher herds with alarms based on the total antibiotic consumption data obtained prior to, during, and after becoming positive for Porcine pleuropneumonia (caused by *Actinobacillus pleuropneumoniae*) (month=0).

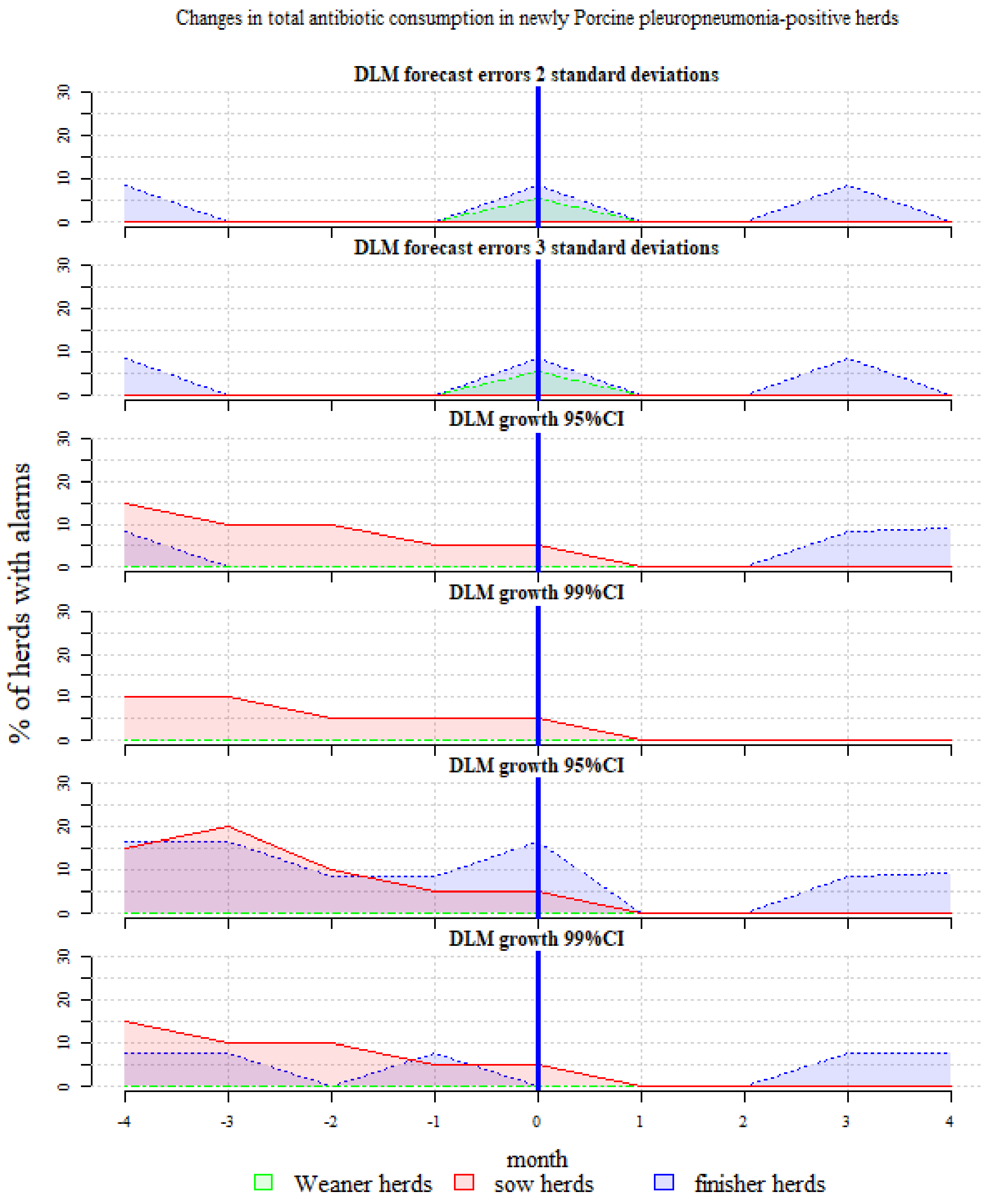

Fig F. Percentage of weaner, sow, and finisher herds with alarms based on antibiotic consumption for respiratory diseases data obtained prior to, during, and after becoming positive for Porcine pleuropneumonia (caused by *Actinobacillus pleuropneumoniae*) (month=0).

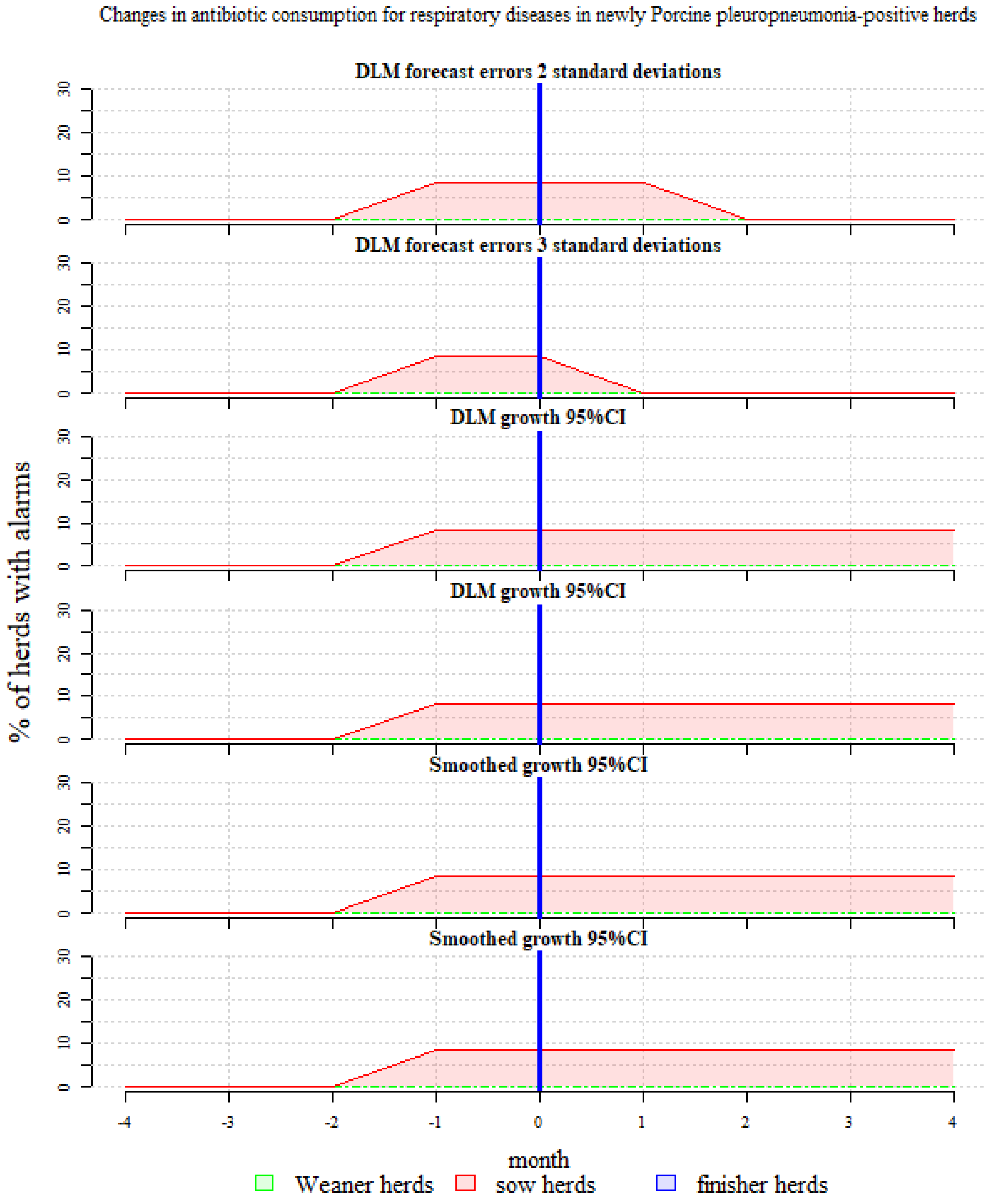

Supplement: S3 Appendix — The alarms were generated based on Shewhart control charts (using 2σ and 3σ thresholds) applied to standardized forecast errors, and on the growth component (based on 95% CI and 99% CI) obtained from a Dynamic Linear Model (DLM) with a Kalman filter performing prospective analysis, as well as the growth component (based on 95% CI and 99% CI) obtained from a smoother performing retrospective analysis. The number of herds included in these calculations can be found in Table 1. Fig A. Percentage of weaner, sow, and finisher herds with alarms based on mortality data obtained prior to, during, and after becoming positive for Porcine Reproductive and Respiratory Syndrome (PRRS) (month = 0). Fig B. Percentage of weaner, sow, and finisher herds with alarms based on the total antibiotic consumption data obtained prior to, during, and after becoming positive for Porcine Reproductive and Respiratory Syndrome (PRRS) (month = 0). Fig C. Percentage of weaner, sow, and finisher herds with alarms based on antibiotic consumption for respiratory diseases data obtained prior to, during, and after becoming positive for Porcine Reproductive and Respiratory Syndrome (PRRS) (month = 0). Fig D. Percentage of weaner, sow, and finisher herds with alarms based on mortality data obtained prior to, during, and after becoming positive for Porcine pleuropneumonia (caused by Actinobacillus pleuropneumoniae) (month = 0). Fig E. Percentage of weaner, sow, and finisher herds with alarms based on the total antibiotic consumption data obtained prior to, during, and after becoming positive for Porcine pleuropneumonia (caused by Actinobacillus pleuropneumoniae) (month = 0). Fig F. Percentage of weaner, sow, and finisher herds with alarms based on antibiotic consumption for respiratory diseases data obtained prior to, during, and after becoming positive for Porcine pleuropneumonia (caused by Actinobacillus pleuropneumoniae) (month = 0). (PDF) [file pone.0223250.s003.pdf]
